# Mutation burden of narrowband ultraviolet B phototherapy (NB-UVB) in human skin: relevance to NB-UVB lifetime exposures and skin cancer surveillance

**DOI:** 10.1093/bjd/ljaf173

**Published:** 2025-05-03

**Authors:** Joanna C Fowler, Roshan K Sood, George Coltart, Chester Lai, Noeline Nadarajah, John W Holloway, Matthew J J Rose-Zerilli, Brian Diffey, Philip H Jones, Eugene Healy

**Affiliations:** Wellcome Sanger Institute, Wellcome Genome Campus, Hinxton, Cambridge, UK; Wellcome Sanger Institute, Wellcome Genome Campus, Hinxton, Cambridge, UK; Dermatopharmacology, University of Southampton, Southampton General Hospital, Southampton, UK; Dermatology, University Hospital Southampton NHS Foundation Trust, Southampton, UK; Dermatopharmacology, University of Southampton, Southampton General Hospital, Southampton, UK; Dermatology, University Hospital Southampton NHS Foundation Trust, Southampton, UK; Department of Medicine and Therapeutics, Faculty of Medicine, The Chinese University of Hong Kong, Hong Kong SAR, China; Dermatopharmacology, University of Southampton, Southampton General Hospital, Southampton, UK; Human Development and Health, University of Southampton, Southampton General Hospital, Southampton, UK; Institute for Life Sciences, University of Southampton, Southampton, UK; Institute for Life Sciences, University of Southampton, Southampton, UK; Cancer Sciences, University of Southampton, Southampton General Hospital, Southampton, UK; Translational and Clinical Research Institute (Dermatology), Newcastle University, Newcastle upon Tyne, UK; Wellcome Sanger Institute, Wellcome Genome Campus, Hinxton, Cambridge, UK; Department of Oncology, University of Cambridge, Hutchison Research Centre, Cambridge, UK; Dermatopharmacology, University of Southampton, Southampton General Hospital, Southampton, UK; Dermatology, University Hospital Southampton NHS Foundation Trust, Southampton, UK

## Abstract

**Background:**

Ultraviolet radiation (UVR) is used as treatment for psoriasis and other skin diseases, but UVR can induce DNA mutations that may lead to skin cancer development. While skin cancers have been documented in patients treated with phototherapy, a limited number of epidemiological studies have examined the incidence of skin cancer in people receiving narrowband ultraviolet B (NB-UVB) treatment. Information on the mutagenicity of NB-UVB would help inform about the potential skin cancer risks of this treatment.

**Objectives:**

To determine the mutation burden in human skin resulting from a NB-UVB treatment course and to use this data to estimate the total number of NB-UVB exposures whereupon skin cancer surveillance should begin.

**Methods:**

Biopsies of normal skin were obtained before and after a course of NB-UVB from 16 patients with psoriasis. Epidermal DNA was sequenced using nanorate sequencing (NanoSeq) to determine the mutational signatures and mutation burden from NB-UVB.

**Results:**

The NB-UVB treatment course increased the number of mutations in skin. Median increase in mutation burden was 0.55 substitutions per Mb in infrequently sun-exposed (buttock; *n* = 14) skin and 0.89 substitutions per Mb in frequently sun-exposed (forearm; *n* = 10) skin (*P* < 0.001). Change in mutation burden due to NB-UVB ranged from 1.16- to 10.50-fold in buttock skin and from 0.93- to 2.33-fold in forearm skin. This increase was mainly attributable to UVR exposure-linked mutational signatures, SBS7a and SBS7b, with some evidence that mutational burden was related to the genetic background of the individual. Modelling the change in mutation burden from NB-UVB relative to minimal erythema dose (MED) in comparison with average mutation burden in keratinocyte skin cancers allowed estimation of total lifetime exposures at which patients are likely to require skin cancer surveillance; for patients with a MED equal to 2 standard erythemal doses (SEDs), skin cancer surveillance should be offered at 422, 165 and 58 NB-UVB exposures for those receiving low, typical and high levels of sun exposure, respectively.

**Conclusions:**

A treatment course of NB-UVB causes UVR-induced mutations in the healthy skin of patients with psoriasis. Relating mutation burden to MED and sun behaviour habits allows estimation of when to begin skin cancer surveillance according to total lifetime NB-UVB exposures.

## Graphical Abstract

**Graphical Abstract:**
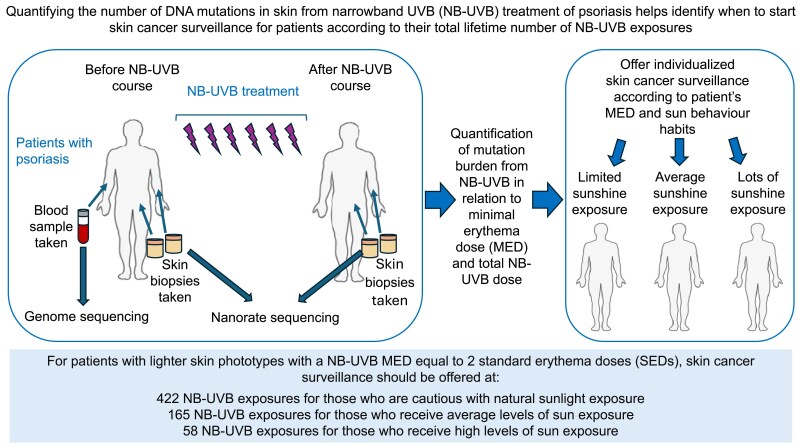

## Teaser

This study used nanorate sequencing (NanoSeq) to quantify the mutation burden in human skin resulting from a course of narrowband ultraviolet B (NB-UVB) treatment. Modelling the change in mutation burden from NB-UVB relative to minimal erythema dose (MED) compared with the mutation burden in keratinocyte cancers allowed for the estimation of total lifetime exposures at which patients are likely to require skin cancer surveillance. For patients with a lighter skin phototype with MED equal to 2 standard erythemal doses, skin cancer surveillance should be offered at 422 exposures for those who rarely expose their skin to sunlight, at 165 exposures for those who receive average levels of sun exposure and at 58 exposures for those who receive high levels of sun exposure.

Linked Article: Paragh and Huss *Br J Dermatol* 2025; **193**:589–590.

What is already known about this topic?

- Narrowband ultraviolet B (NB-UVB) is a type of ultraviolet radiation (UVR) therapy used to treat psoriasis and other skin diseases.
- UVR can cause DNA mutations and the development of skin cancer.
- The number of DNA mutations (i.e. mutation burden) that occur in human skin from a treatment course of NB-UVB is unknown.

What does this study add?

- Nanorate sequencing was used to investigate the number of DNA mutations arising from an NB-UVB treatment course.
- The mutation burden in the healthy skin of patients with psoriasis increased due to NB-UVB in frequently and infrequently sun-exposed skin.
- Modelling of mutation burden in relation to minimal erythema dose, expressed in units of standard erythema dose (SED), allowed estimation of when to start skin cancer surveillance according to total lifetime NB-UVB exposures and patient sun behaviour habits.

What is the translational message?

- For patients with an NB-UVB minimal erythema dose equal to 2 SED, skin cancer surveillance should be offered at 422 NB-UVB exposures for those who are cautious with regard to natural sunlight exposure, at 165 NB-UVB exposures for those who receive typical/average levels of sun exposure and at 58 NB-UVB exposures for those who receive high levels of sun exposure.

Ultraviolet radiation (UVR) can cause DNA mutations in skin and result in the development of skin cancer.^1,2^ However, UVR is used to treat skin diseases, and narrowband ultraviolet B (NB-UVB), wavelength 311 nm, is recommended as a treatment for psoriasis.^3–5^ Despite NB-UVB inducing DNA photoproducts in keratinocytes and being more mutagenic and carcinogenic in mice than broadband UVB, some epidemiological studies have not shown an increased risk of skin cancer from NB-UVB in humans.^6–11^ Conversely, a recent study recorded more skin cancers in patients who received NB-UVB,^12^ and the risk of actinic keratosis also increases significantly for patients with ≥ 200 NB-UVB exposures.^13^ Unfortunately, variability between studies and lack of sufficiently powered prospective studies on patients treated with NB-UVB limits the ability to estimate skin cancer risks following NB-UVB.^10^

In healthy skin, the mutation burden varies with genetic background, age and body site, depending on how often skin is exposed to sunlight;^14^ typical values are > 10-fold lower than those in keratinocyte cancers.^15,16^ Whereas various DNA sequencing techniques (exome, genome, target-enriched) identify mutations in skin cancers, precancers and mutated skin clones,^14,17,18^ they fail to detect mutations in epidermal cells that have not expanded into clones. Nanorate sequencing (NanoSeq) identifies mutations in DNA from single cells to high accuracy, with error rates < 5 per billion base pairs of DNA,^19^ and is ideal for detecting mutations from NB-UVB.

To determine the mutagenic effects of NB-UVB in human skin and estimate the appropriate lifetime number of NB-UVB exposures whereupon skin cancer surveillance would commence, we took biopsies of healthy skin from patients with psoriasis before and after a course of NB-UVB. Following microdissection of epidermis, NanoSeq was performed on epidermal DNA. Comparison of pre-NB-UVB and post-NB-UVB samples allowed quantification of epidermal mutations from NB-UVB, providing insight into the mutagenicity of NB-UVB in human skin. Assessing the number of NB-UVB exposures that would generate a mutation burden equivalent to that in cutaneous squamous cell carcinoma (cSCC) allowed estimation of the number of NB-UVB exposures, according to minimal erythema dose (MED) and sun behaviour habits, regarding when to consider skin cancer surveillance for patients.

## Patients and methods

### Ethical approval and patient samples

Skin biopsies (6-mm punch) from healthy nonlesional skin were obtained with local anaesthesia before and after an NB-UVB course from patients undergoing treatment for psoriasis. Venous blood obtained from each patient allowed comparison of DNA sequences in skin against germline DNA.

### Phototherapy course

Patient age, sex, ethnicity, previous phototherapy, history of skin cancer and pigmentation phenotype were recorded. NB-UVB was administered in upright cabinets, with the dose adjusted according to the MED,^4^ and supervised by healthcare professionals who recorded the treatment doses and number of exposures. To relate UVR dose during phototherapy to natural sunlight exposure, we expressed the MED and cumulative exposure during NB-UVB in standard erythema dose (SED) units. The reason for expressing ultraviolet (UV) doses in SED rather than J cm^–2^ is because the action spectrum for photocarcinogenesis is similar to that for erythema,^20,21^ and so SED is often used as a proxy for carcinogenic-effective UV dose. Equivalence between NB-UVB dose expressed in J cm^–2^ and SED is achieved by weighting the spectral power distribution of a NB-UVB lamp, E(λ), with the action spectrum for erythema,^21^ ε(λ), at each wavelength λ nm and integrating over all UV wavelengths. An NB-UVB dose of 1 J cm^–2^ is then equal to


(1) $$100\times \int_{250}^{400}E(\lambda )\epsilon (\lambda )d\lambda /\int_{250}^{400}E(\lambda )d\lambda =5.7\;SED$$

### Nanorate sequencing

NanoSeq was performed according to Abascal *et al*.^19^ The preparation of DNA for NanoSeq, the principles and methodology of NanoSeq and the mutational signature analysis are provided in Appendix S1 and Table S1 (see Supporting Information).^22^

### Determination of lifetime narrowband ultraviolet B exposures

The average mutation burden of cSCC and basal cell carcinoma (BCC) from exome sequencing data are 50 and 65 substitutions per Mb, respectively.^15,16^ Taking a critical value of 50 substitutions per Mb, we estimated the number of NB-UVB exposures required to achieve this value on generally covered body sites, notably trunk and limbs, at 80 years of age as:


(2) $$\begin{array}{l} Lifetimeno.exposures=M_{exposures}\times \left(50/S_{MED}\right)\\ /\left[M_{dose}+80\times annual\;solar\;exposure\;to\;trunk\;and\;limbs\right] \end{array}$$

*M_exposures_* represents the median number of exposures per course, *M_dose_* the median total dose per course and *S_MED_* is the increase in mutation burden divided by total NB-UVB dose in the treatment course (Δ–mutation burden/dose) for a patient with a given MED, defined by equation 3. While areas of high sun exposure, such as the face, are common sites for keratinocyte cancers, we estimated the risk on trunk and limbs as we assumed the face is normally protected by a UV-opaque visor during phototherapy.

### Polygenic risk scoring pipeline

Full details on polygenic risk score are provided in Appendix S1.

### Cell culture: 8-methoxypsoralen and ultraviolet A

Full details on cell culture are provided in Appendix S1.

## Results

Twenty-one patients with psoriasis were recruited. Biopsies of nonlesional skin before and after the NB-UVB course were taken from 16 patients, whereas pre-NB-UVB but not post-NB-UVB biopsies were obtained from 5 patients. Patients who provided pre-NB-UVB and post-NB-UVB biopsies comprised 6 women and 10 men [median age 33 years (range 20–73)]. According to UK census categories, 13 patients identified as ‘White British’, 1 as ‘White Irish’, 1 as ‘Asian Indian’ and 1 as ‘Asian other’. The patients from whom post-NB-UVB biopsies were not acquired included one woman and four men [median age 50 years (range 21–81)]; four identified as White British and one as White Irish.

Skin biopsies from non-sun-exposed buttock before NB-UVB, and 1–2 cm distant from this after NB-UVB completion, were obtained in 16 cases. In 11 of these patients, skin biopsies were also collected from a frequently sun-exposed dorsal forearm site before and after the NB-UVB course. Following DNA extraction from epidermis, and library preparation, NanoSeq was performed, and paired measurements of mutation burden that passed sequencing quality metrics obtained from 15 patients, including from the buttock in 14 and the forearm in 10 (Table 1). Pre-NB-UVB skin biopsies for all patients were taken before the start of NB-UVB, either the day before or the day of starting NB-UVB therapy. The timing of the second biopsies ranged from 7 to 105 (median 22) days after completion of NB-UVB (Table 2); this was dependent on the patient’s availability to attend for the second biopsy. *In silico* digestion estimates that approximately one-third of the genome is covered by NanoSeq libraries, so although this technique provides accurate information on mutation burden, it is not appropriate to look for mutations in specific genes or driver selection.

**Table 1:** Details of patients who provided skin biopsies before and after a course of narrowband ultraviolet B treatment

Patient	Sex	Age (years)	Ethnicity^a^	Hair colour	Eye colour (peripheral/peripupillary)	Colour of darkest previous suntan from repeated sun exposure	History of freckles (including after sun exposure)	Previous UVR therapy
1	M	26	White Irish	Light brown	Blue/blue	Light golden	Yes	No
2	M	37	Asian other	Black	Brown/brown	Dark brown	No	No
3	F	30	White British	Light brown	Grey/green	Medium brown	Yes	Yes (UVB)
4	M	50	Asian Indian	Black	Tan/brown	Dark brown	No	Yes (UVB)
6	F	20	White British	Ash brown	Yellow/tan	Medium brown	No	Yes (NB-UVB)
7	F	24	White British	Medium brown	Blue/grey	Light golden	No	No
8	M	73	White British	Ash blonde	Grey/tan	Medium brown	No	Yes (PUVA, NB-UVB)
10	M	55	White British	Medium brown	Grey/green	Dark brown	No	Yes (NB-UVB)
11	F	26	White British	Light brown	Blue/grey	Light golden	No	No
12	M	32	White British	Dark brown	Tan/brown	Light golden	Yes	Yes (NB-UVB)
14	M	60	White British	Dark brown	Grey/green	Dark brown	No	No
15	F	59	White British	Dark brown	Grey/blue	Medium brown	Yes	Yes (NB-UVB)
17	M	52	White British	Dark brown	Brown/brown	Dark brown	Yes	Yes (NB-UVB)
18	F	34	White British	Dark brown	Grey/tan	Medium brown	No	Yes (NB-UVB)
19	M	26	White British	Ash blonde	Green/tan	Light golden	Yes	Yes (type unknown)

F, female; M, male; NB-UVB, narrowband UVB; PUVA, psoralen + ultraviolet A; UVB, ultraviolet B; UVR, ultraviolet radiation. ^a^UK census categories.

**Table 2:** Narrowband ultraviolet B (NB-UVB) course parameters and mutation burdens in buttock skin and forearm skin

Patient	MED (SED)^a,b^	No. exposures in this NB-UVB course	Total dose in this NB-UVB course SED^a^ (MED J cm^–2^)	Mutation burden pre-NB-UVB (substitutions per Mb)	Mutation burden post-NB-UVB (substitutions per Mb)	Duration between final NB-UVB exposure and second biopsy (days)	Fold change in mutation burden in skin from baseline	Δ–mutation burden (substitutions per Mb per SED)
Buttock skin								
1	2.9 (0.5)	33	165 (28.89)	0.072	0.685	34	9.55	0.0037
2	5.1 (0.9)	30	756 (132.66)	0.098	0.260	11	2.65	0.0002
3	1.7 (0.3)	23	151 (26.44)	0.575	1.576	28	2.74	0.0066
4	4.0 (0.7)	14	84 (14.77)	0.195	0.225	82	1.16	0.0004
6	2.9 (0.5)	28	183 (32.18)	0.434	0.799	13	1.84	0.0020
7	2.3 (0.4)	35	375 (65.81)	0.081	0.853	10	10.50	0.0021
8	1.7 (0.3)	29	237 (41.60)	1.174	1.613	22	1.37	0.0019
10	2.9 (0.5)	28	427 (74.92)	0.830	1.219	22	1.47	0.0009
11	1.7 (0.3)	34	217 (38.06)	0.207	0.679	14	3.28	0.0022
12	2.9 (0.5)	30	192 (33.67)	0.801	1.622	61	2.02	0.0043
15	1.7 (0.3)	26	219 (38.38)	2.740	3.599	105	1.31	0.0039
17	1.7 (0.3)	35	529 (92.77)	1.478	2.191	105	1.48	0.0013
18	1.7 (0.3)	28	224 (39.36)	1.065	1.836	98	1.72	0.0034
19	2.9 (0.5)	28	419 (73.45)	0.470	0.966	12	2.05	0.0012
Forearm skin								
6	2.9 (0.5)	28	183 (32.18)	0.950	2.217	13	2.33	0.0069
7	2.3 (0.4)	35	375 (65.81)	1.996	2.584	10	1.29	0.0016
8	1.7 (0.3)	29	237 (41.60)	5.199	6.681	22	1.29	0.0063
10	2.9 (0.5)	28	427 (74.92)	4.069	5.198	22	1.28	0.0026
11	1.7 (0.3)	34	217 (38.06)	2.547	2.372	14	0.93	–0.0008
12	2.9 (0.5)	30	192 (33.67)	4.967	5.793	61	1.17	0.0043
14	5.1 (0.9)	21	128 (22.42)	2.337	2.337	7	1.00	0.0000
17	1.7 (0.3)	35	529 (92.77)	7.498	10.591	105	1.41	0.0058
18	1.7 (0.3)	28	224 (39.36)	3.578	4.524	98	1.26	0.0042
19	2.9 (0.5)	28	419 (73.45)	2.688	3.024	12	1.12	0.0008

MED, minimal erythema dose; ^a^1 J cm^–2^ of NB-UVB is equivalent to an erythemally effective dose of 5.7 SED; ^b^MED (J cm^–2^) provided in parentheses.

Information on natural hair colour, eye colour, freckling, darkest tan the patient ever obtained following sun exposure and previous phototherapy is provided in Table 1. No patient had a history of skin cancer. MED ranged from 1.7 to 5.1 SED (median 2.9), number of NB-UVB exposures during treatment ranged from 14 to 35 (median 28) and total NB-UVB dose varied from 84 to 756 SED (median 219) (Table 2). Two patients had a family history of skin cancer (patient 1’s grandfather had skin cancer of unknown type and patient 3’s aunt had melanoma). Four patients had previously received immunosuppressive treatment (patients 2, 15 and 17 had taken methotrexate for 27, 18 and 28 months, respectively; patient 4 had received multiple courses of oral steroids for ulcerative colitis several years earlier). No patient had immunosuppressive treatment in the 6 months before or during the NB-UVB course. Four patients were taking photosensitizing medication at the start of and during the NB-UVB course (patient 4, lansoprazole; patient 7, amitriptyline; patient 8, simvastatin; patient 17, acitretin).

**Figure 1:**
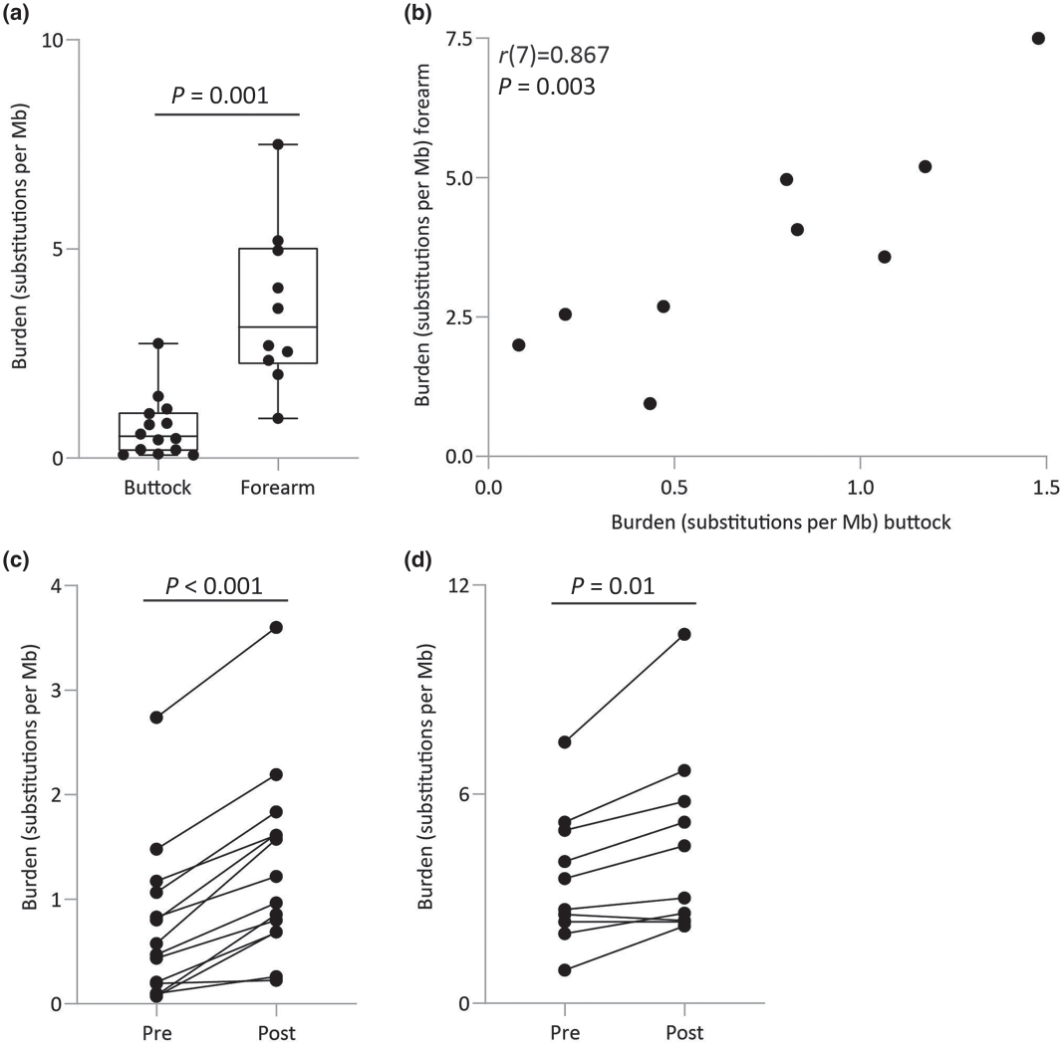
(a) Pretreatment mutational burden for buttock and forearm samples calculated by nanorate sequencing. Each point represents a patient (buttock, *n* = 14; forearm, *n* = 10). *P*-value obtained from a Kolmogorov–Smirnov test. (b) Pearson’s correlation analysis of pretreatment mutational burdens between matched forearm and buttock samples. Each point represents a patient (*n* = 9). (c, d) Mutational burden in pre- and post-narrowband ultraviolet B-treated samples in (c) buttock and (d) forearm samples. Lines link matched samples from the same patient (buttock, *n* = 14; forearm, *n* = 10). *P*-value was obtained from a two-tailed Wilcoxon signed-rank test.

Prior to NB-UVB, the mutation burden was lower in buttock (median 0.52 substitutions per Mb, *n* = 14 patients) than in forearm (3.13 substitutions per Mb, *n* = 10 patients) skin (*P* = 0.001, Kolmogorov–Smirnov test; Figure 1a). Pretreatment mutation burden ranged from 0.07 to 2.74 substitutions per Mb in buttock skin and from 0.95 to 7.5 substitutions per Mb in forearm skin (Table 2), but they were positively correlated (Pearson’s) within patients [*r*(7) = 0.867, *P* = 0.003; Figure 1b]. The mutation burden in buttock skin was higher in patients who previously received phototherapy (range 0.195–2.740 substitutions per Mb, *n* = 10 patients) than in those who had not had phototherapy previously (range 0.072–0.207 substitutions per Mb, *n* = 4 patients) (*P* = 0.01, Kolmogorov–Smirnov test). NB-UVB treatment resulted in increased mutation burdens in buttock (median post-treatment 1.09 substitutions per Mb, *n* = 14; *P* < 0.001, Wilcoxon signed-rank test) and forearm (median post-treatment 3.77 substitutions per Mb, *n* = 10; *P* = 0.01, Wilcoxon signed-rank test) skin (Figure 1c, d). Change in mutation burden within a patient due to NB-UVB ranged from 1.16- to 10.50-fold in buttock and from 0.93- to 2.33-fold in forearm skin (Table 2).

**Figure 2:**
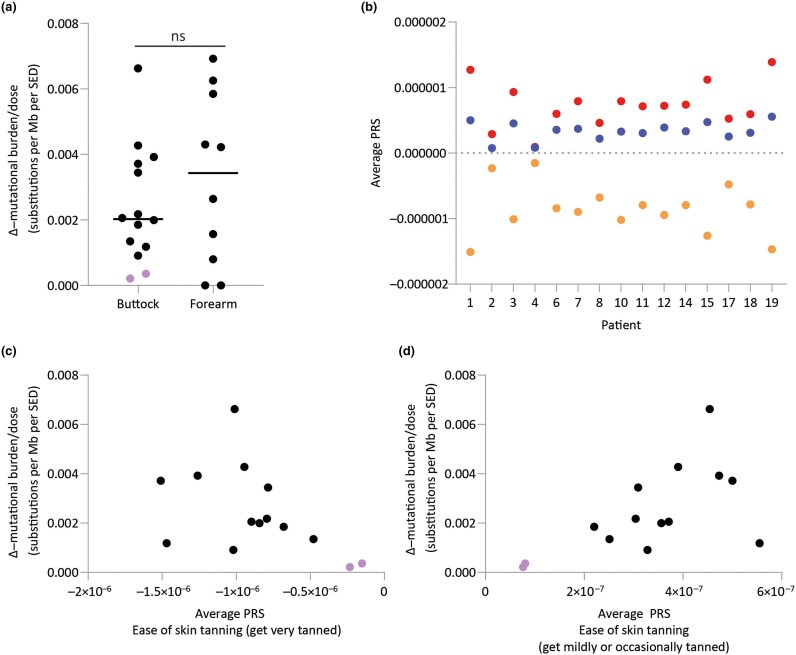
(a) Change in mutational burden between pre- and post-treatment buttock samples (*n* = 14) and forearm samples (*n* = 10) normalized to total narrowband ultraviolet B (NB-UVB) dose received over the course of treatment. Highlighted samples indicate patients of Asian heritage. (b) Average polygenic risk scores (PRS) for ease of skin tanning as assessed by single nucleotide polymorphisms (SNPs) detailed in Tanigawa *et al*. (see Appendix S1; *n* = 15 patients).^38^ SNPs associated with propensity to ‘never tan, only burn’ (PGS001247 red) comprises 3159 variants; SNPs associated with a tendency for a mild tan (PGS001244 blue) includes 958 variants; and the ability to become very tanned (PGS001246 orange) consists of 4130 variants. (c, d) Change in burden normalized to total NB-UVB in buttock samples plotted against average PRS reflecting the risk of (c) getting very tanned or (d) getting mildly or occasionally tanned. Highlighted samples indicate patients of Asian heritage. Ave, average; ns, not statistically significant; SED, standard erythema dose.

The increase in mutation burden varied within buttock skin from 0.03 (patient 4) to 1.00 (patient 3) substitutions per Mb. Therefore, we normalized the increase in mutation burden for total NB-UVB dose received by each patient (Δ–mutation burden/dose). Δ–mutation burden/dose for buttock skin ranged from 0.0002 to 0.0066 (median 0.0021) substitutions per Mb per SED (Figure 2a, Table 2) and from 0.0000 to 0.0069 (median 0.0034) substitutions per Mb per SED in forearm skin (Figure 2a, Table 2). The two lowest Δ–mutation burden/dose values for buttock skin were found in Asian patients (median 0.0003 substitutions per Mb per SED); for other patients, Δ–mutation burden/dose values ranged from 0.0009 to 0.0066 (median 0.0021) substitutions per Mb per SED (Figure 2a). Comparison of Δ–mutation burden/dose between buttock and forearm skin showed no significant difference (*P* = 0.55, Mann–Whitney *U* test). No association was seen between Δ–mutation burden/dose and duration from final NB-UVB exposure to the second biopsy in buttock or forearm skin (*P* = 0.23 and *P* = 0.13, respectively; Spearman’s rank test). A positive association was noted between increasing age and baseline mutational burden pre-NB-UVB in buttock and forearm skin (*P* = 0.01 and *P* < 0.05, respectively; Spearman’s rank test). No association was observed between age and Δ–mutation burden/dose in buttock or forearm skin (*P* = 0.42 and *P* = 0.88, respectively; Spearman’s rank test). However, Δ–mutation burden/dose in buttock, and in buttock and forearm skin combined, was negatively associated with MED [*P* = 0.02 and *P* = 0.007, respectively; Spearman’s rank test (in the latter analysis, patient 11’s forearm skin was omitted due to lack of biologic plausibility that a phototherapy course would reduce the number of mutations in the skin)].

As Fitzpatrick skin phototyping is inaccurate in approximately 40–60% of people,^23,24^ we asked patients about the darkest suntan they had ever developed from sun exposure. Fold change in mutation burden in buttock skin of those reporting light golden vs. those reporting a medium- or dark-brown tan ranged from 2.02 to 10.5 (median 3.28) and from 1.16 to 2.74 (median 1.48), respectively (*P* = 0.01, Mann–Whitney *U* test). Therefore, we examined the variation in germline genetic loci that predispose to tanning from UVR in the included patients. Polygenic risk scores estimate genetic predisposition to quantitative traits – in this case the ability to tan – by aggregating the effects across multiple genetic variants,^25^ including 3159 variants for propensity to ‘never tan, only burn’, 958 variants for ‘mildly or occasionally tanned’ and 4130 variants for ‘very tanned’ (Table S2; see Supporting Information). Polygenic risk scores for tanning varied across the cohort, but both Asian participants with low Δ–mutation burden/dose values had scores indicating a higher propensity to become very tanned (Figure 2b–d).

**Figure 3:**
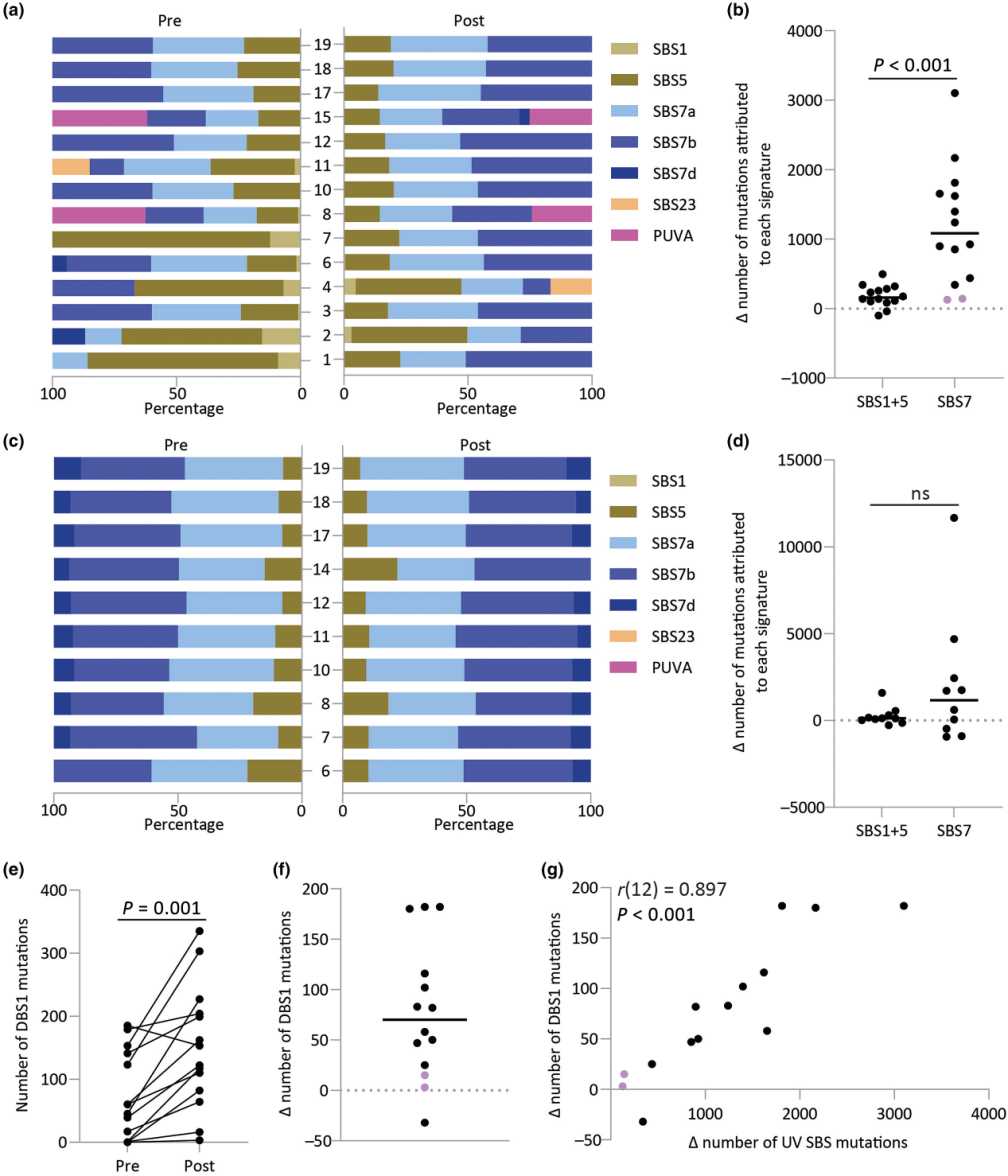
(a) Assignment of COSMIC signatures plus psoralen + ultraviolet A (PUVA) signature described by Olafsson *et al*. by Sigprofiler using SBS288 context.^28^ The left-hand panel denotes signatures called from pretreatment buttock samples; the right-hand side shows the matched post-treatment samples (*n* = 14). (b) Change in number of mutations after treatment in matched buttock samples attributable to either ultraviolet radiation (UVR) exposure (SBS7a, SBS7b, SBS7d) or ageing (SBS1 and SBS5; *n* = 14). Points highlighted in purple indicate patients of Asian heritage. The *P*-value was obtained from a Mann–Whitney *U* test. (c) Assignment of COSMIC signatures plus PUVA signature described by Olafsson *et al*. by Sigprofiler using SBS288 context.^28^ The left-hand panel denotes signatures called from pretreatment forearm samples; the right-hand side shows the matched post-treatment samples (*n* = 10). (d) Change in number of mutations after treatment in matched forearm samples attributable to either UVR exposure (SBS7a, SBS7b, SBS7d) or ageing (SBS1 and SBS5; *n* = 10). The *P*-value was obtained from a Mann–Whitney *U* test. (e) Number of DBS1 (primarily CC > TT) mutations as assessed by Sigprofiler in pre- and post-treatment buttock samples (*n* = 14). The *P*-value was obtained from a Wilcoxon signed rank test. (f) Change in number of mutations attributed to DBS1 between the pre- and post-treatment buttock samples (*n* = 14). Points highlighted in purple indicate patients of Asian heritage. (g) Pearson’s correlation analysis of change in the number of double base substitutions attributed to DBS1 and change in the number of single base substitutions associated with UVR signatures (SBS7) in buttock samples (*n* = 14). Each point represents a patient. Points highlighted in purple indicate patients of Asian heritage. ns, not statistically significant.

Next, we investigated mutational signatures in pre-NB-UVB and post-NB-UVB samples. UVR signatures SBS7a and SBS7b were not only identified in all post-NB-UVB buttock and forearm skin samples, but were also present in all pre-NB-UVB forearm and most pre-NB-UVB buttock samples (Figure 3a–d). Patients with low SBS7 mutational burden (patients 1, 2, 4 and 7) in pretreatment buttock skin showed a high proportion of mutations attributable to ageing signatures 1 and 5 (Figure 3a); however, after treatment, their buttock skin had a substantial proportion of SBS7a and SBS7b mutations. Overall, the change in mutation burden in buttock skin from NB-UVB attributed to each signature was predominately due to UVR (SBS7a–d), except in Asian patients (*P* < 0.001, Mann–Whitney *U* test; Figure 3b).^22^

In patients 8 and 15, we noted a mutational signature similar to that reportedly associated with historical psoralen + UVA (PUVA) treatment [Figure 3a; Figure S1 (see Supporting Information)].^22,26–28^ We validated this signature by treating HaCaT keratinocytes with 8-methoxypsoralen (8-MOP) and UVA, and analysing with NanoSeq (Figure S2a, b; see Supporting Information). *De novo* signature analysis revealed two signatures, SBS288A and SBS288B, the latter of which was associated with combined 8-MOP and UVA (Figure S2c). SBS288B includes the APOBEC signature (SBS2 and SBS13), with additional T > A, T > C and T > G changes consistent with the reported PUVA signature (Figure S2d–f).^22,26–28^ While patient 8 had PUVA in 2016, patient 15 denied having PUVA but had used suntan products regularly since the 1970s, and her PUVA signature may have reflected previous use of psoralen-containing suntan preparations.^29^ The PUVA signature was not detected in patient 8’s forearm skin, which was dominated by UVR (SBS7a–d) mutations pre-NB-UVB and post-NB-UVB (Figure 3c).

A significant increase in the double base substitution signature DBS1 (primarily denoting CC > TT mutations) was seen in buttock skin (Figure 3e, f), as expected from UVR exposure (*P* = 0.001; Wilcoxon signed-rank test).^22^ In all cases except one, the number of DSB1 mutations in buttock skin had increased after NB-UVB; in patient 10, DSB1 reduced by 17.3%, despite a 46.8% increase in single base substitutions (SBS) per Mb mutation burden in the same skin samples. Change in the number of DSB1 mutations was positively correlated with change in the number of SBS mutations attributed to UVR (SBS7) in the buttock [*r*(12) = 0.897, *P* < 0.001; Figure 3g]. The lowest number of DSB1 mutations were found in the pre- and post-NB-UVB samples from Asian patients.

**Figure 4:**
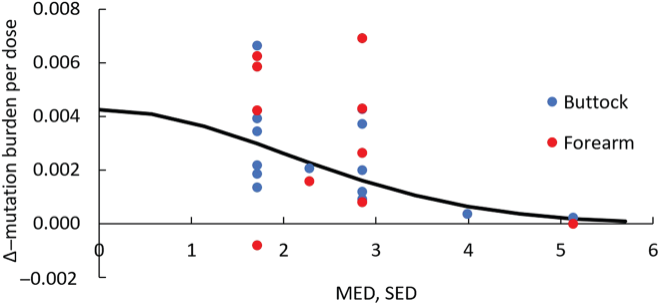
Increase in mutation burden per dose of narrowband ultraviolet B (NB-UVB) in relation to minimal erythema dose (MED) expressed as standard erythema dose (SED) for each patient. The solid curve is the regression fit of the relationship expressed by equation 3; the red value beneath the *x*-axis (patient 11’s forearm skin) was omitted from the regression fit due to the lack of biologic plausibility that a course of NB-UVB would reduce the number of mutations in skin. Δ–mutation burden/dose is expressed as substitutions per Mb per SED.

The Δ–mutation burden/dose in buttock and/or forearm skin for each patient plotted against their MED is shown in Figure 4. The solid curve is an expression of the formula:


(3) $$S_{MED}=A.\;exp(-b.MED^{2})$$

where *A* = 0.0042 and *b* = 0.12 ± 0.03, obtained using a linear regression fit of log_e_(Δ–mutation burden/dose) vs. *MED*^2^.

Estimates of personal solar UV exposure are normally obtained by direct measurement using UV-sensitive film badges or electronic dosimeters generally worn on the wrist. Results from studies in mid-latitudes indicate broadly that people receive an annual exposure of approximately 200 SED mainly from summer weekend and holiday exposure, principally to their hands, forearms and face.^30^ Our data indicate that a typical NB-UVB course results in a cumulative whole-body dose of 200–300 SED (Table 2), such that the trunk and limbs receive equivalent to that obtained by exposed skin sites in 1–2 years’ sun exposure.

However, at the population level annual exposure can vary enormously. Daily sun exposure on vulnerable body sites of someone engaging in sun-seeking behaviour in areas of high insolation (UV index up to 11) can lead to exposures of 25 SED over 1 day,^31^ and sun protection is necessary to prevent marked sunburn. It is also possible to take a beach holiday that results in much lower exposure; Narbutt *et al*. reported on a week-long beach holiday in Tenerife during late March (mean UV index around 9 at solar noon) where the mean daily exposure averaged over 40 participants was 6 SED.^32^ These data imply that a typical NB-UVB course equates to an exposure to the trunk and limbs of an annual 7-day beach holiday for 2–6 years.

Therefore, we can estimate the lifetime number of NB-UVB exposures (equation 2) for people who are particularly cautious in the sun, typical people (neither cautious nor enthusiastic) and people who are enthusiastic for sun exposure, where we assume annual solar UV exposure to the (normally covered) trunk and limbs throughout their lives is 10 SED, 30 SED and 90 SED, respectively. Based on these calculations, the number of NB-UVB exposures, with 95% confidence intervals, required to achieve a mutation burden of 50 substitutions per Mb are provided in Table 3.

**Table 3:** Number of lifetime narrowband ultraviolet B (NB-UVB) exposures estimated to result in skin cancer after 80 years of the same annual sun exposure, in relation to minimal erythema dose (MED) and sun behaviour habits, based on Δ–mutation burden/dose mutation burden in buttock and/or forearm skin for each patient plotted against their MED

Attitude to sun exposure	Cautious	Typical	Enthusiastic
Annual sun exposure to trunk and limbs	10 SED	30 SED	90 SED
NB-UVB MED on buttock (J cm^–2^)^a^	No. of NB-UVB exposures (95% CI)		
0.2 (1.1)	292 (220–370)	114 (88–144)	40 (32–54)
0.3 (1.7)	364 (302–440)	142 (117–172)	50 (42–59)
0.35 (2.0)	422 (365–487)	165 (137–194)	58 (49–67)
0.4 (2.3)	497 (455–547)	194 (178–212)	69 (63–75)
0.5 (2.9)	742 (731–752)	290 (285–293)	102 (101–104)
0.6 (3.4)	1211 (1021–1415)	473 (403–548)	167 (150–194)
0.7 (4.0)	2158 (1571–3122)	842 (632–1220)	298 (202–404)
0.8 (4.6)	4206 (2537–6997)	1642 (855–2635)	580 (307–1036)
0.9 (5.1)	8959 (4775–16 610)	3497 (1821–10 211)	1236 (675–3446)
1.0 (5.7)	20 858 (7595–59 699)	8141 (3449–24 333)	2878 (1066–5666)

CI, confidence interval. ^a^Standard erythema dose (SED) in parentheses.

## Discussion

This study shows that an NB-UVB treatment course for psoriasis significantly increases the mutation burden in infrequently sun-exposed (buttock) and frequently sun-exposed (forearm) skin. Attributing this mutation burden increase to NB-UVB is most reliable in buttock skin, because forearm skin may be exposed to sunlight in addition to UVR from therapy. The fact that patients had their second biopsy taken up to 105 days after the completion of NB-UVB and a lack of association between Δ–mutation burden/dose and this duration after NB-UVB suggests the mutations remain in the epidermis beyond the epidermal turnover time. Additionally, the significantly higher mutation burden in buttock skin of patients who received phototherapy previously indicates many mutations from phototherapy remain in the skin over the long term. Furthermore, the observation of a PUVA signature in two patients suggests that NanoSeq is picking up mutations that have been present in the epidermis for many years. Armstrong and Kricker documented a linear relationship between the age-standardized incidence of SCC and BCC vs. solar UVR measurement,^33^ and Rivas *et al.* noted a linear association between skin cancer rates per 100 000 persons and accumulated UV solar index,^34^ suggesting that a linear relationship exists between total UVR dose and skin cancer. Moreover, we evaluated which model (linear, power law, exponential) best represented our data on mutation burden in relation to age for the pre-NB-UVB and post-NB-UVB mutation burdens, and the goodness of fit (*R^2^*) showed a linear model was best. Skin cancer arises from accumulation of UVR-induced somatic genetic mutations,^15,16,18^ and the aforementioned linear observations suggest that cumulative UVR mutation burden is linked in a linear fashion to skin cancer risk.^33,34^

The increase in mutation burden from an NB-UVB course provides insight into the risk of keratinocyte neoplasia from NB-UVB, based on average mutation burdens of cSCC and BCC being 50 and 65 substitutions per Mb, respectively.^15,16^ A previous estimate of the maximum number of NB-UVB exposures by Diffey,^35^ assuming that NB-UVB is as equally carcinogenic as sunlight, indicated a maximum of 500 exposures for risk-averse individuals. Additionally, British Association of Dermatologists and British Photodermatology Group guidelines for narrowband ultraviolet B phototherapy recommend ‘offer[ing] skin cancer surveillance at appropriate intervals to people identified as having received more than 500 whole-body NB-UVB treatments’.^4^ However, the mutation burden in our study allows for an estimation of the number of NB-UVB exposures relative to MED for patients according to their sun behaviour habits. The total number of exposures that can be tolerated decreases with increasing sun sensitivity (lower MED) and a person’s propensity for previous sun exposure to normally covered body sites. For people with lighter skin phototypes, the MED is typically 2.0–2.5 SED,^36^ and (from Table 3) a lifetime number of approximately 422 exposures for sun-sensitive individuals (MED 0.35 J cm^–2^ or 2 SED) who are risk averse or cautious in the sun would be estimated to cause skin cancer, suggesting that skin cancer surveillance should start around (or before) this number of NB-UVB exposures.

Our results also suggest that skin cancer surveillance should start at lower numbers of NB-UVB exposures for people with lighter skin phototypes who are exposed to more sunshine; for example, around 165 exposures for patients with average levels and 58 exposures for individuals with high levels of sun exposure. Moreover, the data suggest that skin cancer surveillance starts earlier than 500 NB-UVB exposures in patients with MED ≤ 3 SED and average sun exposure, and for those with MED ≤ 4 SED with higher amounts of sun exposure. However, given the variability of mutation burden for a given MED, these estimates should be regarded as simply a guide. Some clinicians may consider an estimation based on 80 years’ sun exposure less relevant to certain patients; therefore, we have provided the number of lifetime NB-UVB exposures estimated to cause skin cancer after 60 and 40 years of the same annual sun exposure in Tables S3 and S4 (see Supporting Information). These tables indicate that skin cancer surveillance should start before 500 NB-UVB exposures for many of these patients. Therefore, it is likely that previous recommendations on skin cancer surveillance will need to be reconsidered for individuals who receive NB-UVB treatment for psoriasis.

The limitations of this study include the small sample size of patients recruited from a single dermatology centre, variability in mutation burden in buttock and forearm skin for a given MED and variability in the Δ–mutation burden/dose. We have previously reported on clones of mutated cells in sun-exposed human skin,^17,18,37^ but NanoSeq is not subject to biases such as clonal expansion because it sequences single molecules of DNA, giving a true estimate of the number of mutations in skin, independently of the presence or absence of mutated clones. While we do not know which NB-UVB-induced mutations will undergo clonal expansion, or which NB-UVB mutations are in epidermal stem cells, our findings demonstrate the feasibility of employing NanoSeq to determine mutation burden from a NB-UVB course and provide a rationale for personalizing skin cancer surveillance for patients receiving NB-UVB according to their MED and exposure to natural sunlight.

## Supplementary Material

ljaf173_Supplementary_Data

## Data Availability

The sequencing datasets generated during the study have been submitted to the European Genome-Phenome Archive (EGA) with the following dataset accession numbers: whole genome sequencing EGAD0000101525; NanoSeq EGAD00001015249. The HaCaT sequencing dataset is available from the European Nucleotide Archive (ENA) with accession number ERP156525.
