# Supplementary material for: Mutation burden of narrowband ultraviolet B phototherapy (NB-UVB) in human skin: relevance to NB-UVB lifetime exposures and skin cancer surveillance
Source: Br J Dermatol. 2025 May 3;193(4):718–28. doi: 10.1093/bjd/ljaf173 (PMC12448954; doi:10.1093/bjd/ljaf173)
Supplement: ljaf173_Supplementary_Data [file ljaf173_supplementary_data.zip › Supporting Information_Edited.docx]

**Appendix S1**

**Supplementary methods**

**Preparation of DNA for NanoSeq**

Skin samples were fixed in PAXgene (Qiagen) and embedded in paraffin. 16µm sections were cut, deparaffinised in xylene, washed in graded ethanols and phosphate buffered saline before haematoxylin and eosin staining. Slides were then rinsed in water, 70% ethanol, 100% ethanol, and air-dried. Dermis was scraped away, and DNA was extracted from three 16µm epidermal sections per patient using the Arcturus Picopure kit (Applied Biosystems). Blood DNA was extracted using the QIAmp DNA mini kit (Qiagen).

**Principles and methodology of Nanoseq**

Nanoseq is a variant of duplex sequencing technology^1^. In the current study, we extracted DNA from the epidermis of 3x 16um paraffin-embedded skin sections. DNA was fragmented by enzymatic digestion, A-tailed and adaptors ligated. Nanoseq sequences the third of the genome flanking the restriction enzyme recognition sites. The regions sequenced are consistent between samples and are sufficient to allow accurate quantification of mutation burden and mutational signatures. After digestion, enzymatic fragments are amplified and sequenced. 0.3 fmol of library from each sample was put forward for sequencing. Pooling of sequenced reads from fragments from both strands allows removal of PCR and sequencing artefacts as genuine somatic variants will occur in all fragments from both strands. In consequence, Nanoseq has an estimated error rate of less 5x10^-9^ errors/bp. Germline mutations were identified in whole genome sequencing from the same patient and were removed. Here we used Nanoseq to sequence an average of 2x10^9^ bases per sample giving an average duplex coverage of 0.64 (Table S1).

50ng DNA was used for dupseq library preparation. Briefly, DNA was digested with mung bean nuclease, A-tailed, repaired and tagged. 0.3fmol of indexed tagged library were sequenced with 14 PCR cycles before quantifying and sequencing on Novaseq6000 (Illumina) with 150bp paired-end reads. 30x coverage whole genome sequence of blood from the same patient was used as germline control for calling SNPs and indels. For HaCaT sequencing, 4fmol indexed-tagged library from untreated cells and 10 PCR cycles was used to call germline SNPs. Contamination from unrelated individuals was assessed using verifyBAMID; samples showing a value >0.005 were excluded from the analysis. Sequencing metrics and duplex coverage is provided in Table S1. For SNV and indels, only calls passing all defined filters (https://github.com/cancerit/NanoSeq) were used.

**Mutational signature analysis**

Mutational spectra and signatures are described using the PCAWG Mutational Signatures notation^2^. COSMIC signature definitions (v3.2) (https://cancer.sanger.ac.uk/signatures/sbs/) were used for Single Base Substitutions (SBS), and Double base Substitutions (DBS) signature classification using SigProfiler packages MatrixGenerator (v1.2.12), Extractor (v1.1.12), Assignment (v0.0.13), Plotting (1.2.2). Frequency of mutations within each trinucleotide context was calculated using SigProfiler within the SBS288 context^2^.

**Polygenic risk scoring pipeline**

Polygenic risk score for tanning was performed using the Polygenic Score Catalog (PGSC), and accompanying PGSC-calc package (v1.3.0), with nextflow (v22.04.5)^~~2~~3^. Risk scores were calculated for EFO terms EFO_0004279 (Suntan). A VCF file of joint called germline variants was produced using best practices guidelines for GATK Haplotype caller (4.3.0.0)^~~3~~4^. 14 polygenic score files were found for these EFO terms split across 3 publications. The Tanigawa et al.^~~4~~5^ publication contained 4 scoring files for tanning-response matches well above the set minimum match fraction of 60% between the score file loci and VCF files24.

**Cell Culture; 8-MOP and UVA**

HaCaT keratinocytes were cultured in DMEM (Sigma) with 10% fetal bovine serum (FBS, Thermo Fisher). Prior to UV, cells were changed to media containing 0.078µM or 0.156µM 8-methoxypsoralen (8-MOP, Sigma), previously published as an effective dose^~~5~~6^. After 30mins 8-MOP treatment, media was removed, and cells placed in PBS and irradiated with 0.6J/cm^2^ UVA (UV-2, Tyler Research Corporation). After irradiation, PBS was removed and cells cultured in DMEM with 10%FBS. Cells were exposed to 8-MOP and subsequent UVA for 10 occasions, averaging once every three days. After this treatment course, cells were trypsinised and DNA extracted using the QIAmp DNA microkit protocol (Qiagen). Regular mycoplasma testing of cells was conducted using PCR, as per Young et al, 2010^~~6~~7^.

**REFERENCES FOR SUPPLEMENTARY METHODS**

1. Abascal F , Harvey LMR , Mitchell E *et al*. Somatic mutation landscapes at single-molecule resolution. Nature 2021;593(7859):405-410. doi: 10.1038/s41586-021-03477-4.

2. Alexandrov LB, Kim J, Haradhvala NJ *et al*. The repertoire of mutational signatures in human cancer. Nature 2020; 578: 94-101. doi: 10.1038/s41586-020-1943-3.

3. Lambert SA, Gil L, Jupp S, et al*.* The Polygenic Score Catalog as an open database for reproducibility and systematic evaluation. Nat Genet. 2021;53(4):420-425. doi: 10.1038/s41588-021-00783-5.

4. Poplin R, Ruano-Rubio V, DePristo MA, et al*.* Scaling accurate genetic variant discovery to tens of thousands of samples. bioRxiv. 2018;201178. doi: https://doi.org/10.1101/201178.

5. Tanigawa Y, Qian J, Venkataraman G, et al. Significant sparse polygenic risk scores across 813 traits in UK Biobank. PLoS Genet. 2022;18(3):e1010105. doi: 10.1371/journal.pgen.1010105.

6. Chowdhari S, Saini N. hsa-miR-4516 mediated downregulation of STAT3/CDK6/UBE2N plays a role in PUVA induced apoptosis in keratinocytes. J Cell Physiol. 2014;229(11):1630-1638. doi: 10.1002/jcp.24608.

7. Young L, Sung J, Stacey G, et al. Detection of Mycoplasma in cell cultures. Nat Protoc. 2010;5(5):929-934. doi: 10.1038/nprot.2010.43.

**SUPPLEMENTARY FIGURE LEGENDS**

**Figure S1** (a,b) Signature decomposition of de-novo signatures as assigned by Sigprofiler using the SBS288 context in both buttock (a) and forearm (b). In both cases the left-hand panel shows the proportion of mutations in the pre-treatment samples and the right-hand panel in the post-treatment samples. (c) Mutational profile of de novo signature SBS288B which shows similarities to the PUVA signature described in Olafsson *et al*.^28^

**Figure S2** (a) Experimental outline. Briefly HaCaTs were treated over a 4-week period +/- 8-methoxypsoralen and +/- UVA. DNA was then extracted and sequenced by NanoSeq. Scale bar = 300µm (b) Mutational burden as assessed by NanoSeq of treated HaCaTs. (c) *De novo* mutational analysis of treated HaCaTs by Sigprofiler using SBS288 context. Replicate samples were pooled together to ensure there were sufficient mutations for signature calling. (d,e) Mutational profile of predicted PUVA signature as shown by Olafsson *et al*.^28^ (d), compared to HaCaT treated *de novo* signature SBS288B (e). (f) Comparison of percentage of mutations within all T>A, T>C and T>G contexts from the both the published PUVA signature^28^ and the HaCaT derived SBS288B. r(48)=0.8336 p<0.0001 Pearson correlation.

**SUPPLEMENTARY TABLE LEGENDS**

**Table S1**

Duplex coverage for samples sequenced by NanoSeq

**Table S2**

Average polygenic risk scores for all patients

**Table S3**

Number of lifetime NB-UVB exposures estimated to result in skin cancer after **60** years of the same annual sun exposure, in relation to MED and sun behaviour habits, based on Δ-mutation burden/dose mutation burden in buttock and/or forearm skin for each subject plotted against their MED.

**Table S4**

Number of lifetime NB-UVB exposures estimated to result in skin cancer after **40** years of the same annual sun exposure, in relation to MED and sun behaviour habits, based on Δ-mutation burden/dose mutation burden in buttock and/or forearm skin for each subject plotted against their MED.
