# Supplementary material for: Mutation burden of narrowband ultraviolet B phototherapy (NB-UVB) in human skin: relevance to NB-UVB lifetime exposures and skin cancer surveillance
Source: Br J Dermatol. 2025 May 3;193(4):718–28. doi: 10.1093/bjd/ljaf173 (PMC12448954; doi:10.1093/bjd/ljaf173)
Supplement: ljaf173_Supplementary_Data [file ljaf173_supplementary_data.zip › Tables S3, S4.docx]

**Table S3.** Number of lifetime NB-UVB exposures estimated to result in skin cancer after **60** years of the same annual sun exposure, in relation to MED and sun behaviour habits, based on Δ-mutation burden/dose mutation burden in buttock and/or forearm skin for each subject plotted against their MED.

| **Attitude to sun exposure** | **Cautious** | **Typical** | **Enthusiastic** |
| --- | --- | --- | --- |
| **Annual sun exposure**  **to trunk and limbs** | **10 SED** | **30 SED** | **90 SED** |
| **NB-UVB MED on buttock J/cm^2^ (SED in parentheses)** | **Number of NB-UVB exposures**  **(95% CI in parentheses)** | | |
| 0.2 (1.1) | 362 (277 - 481) | 148 (123 - 198) | 53 (41 - 71) |
| 0.3 (1.7) | 453 (371 - 549) | 184 (153 - 218) | 66 (54 - 79) |
| 0.35 (2.0) | 525 (437 - 605) | 214 (187 - 240) | 77 (67 - 88) |
| 0.4 (2.3) | 618 (549 - 687) | 252 (229 - 274) | 91 (83 - 101) |
| 0.5 (2.9) | 922 (908 - 937) | 376 (370 - 383) | 135 (133 - 137) |
| 0.6 (3.4) | 1504 (1284 - 1721) | 613 (534 - 750) | 220 (197 - 254) |
| 0.7 (4.0) | 2682 (1844 - 3559) | 1092 (821 - 1403) | 393 (301 - 535) |
| 0.8 (4.6) | 5227 (2939 - 9737) | 2128 (1296 - 3485) | 766 (494 - 1340) |
| 0.9 (5.1) | 11132 (4806 - 29527) | 4533 (2234 - 10009) | 1632 (756 - 3227) |
| 1.0 (5.7) | 25918 (10326 - 86855) | 10554 (3456 - 28504) | 3799 (1436 - 11507) |

**Table S4.** Number of lifetime NB-UVB exposures estimated to result in skin cancer after **40** years of the same annual sun exposure, in relation to MED and sun behaviour habits, based on Δ-mutation burden/dose mutation burden in buttock and/or forearm skin for each subject plotted against their MED.

| **Attitude to sun exposure** | **Cautious** | **Typical** | **Enthusiastic** |
| --- | --- | --- | --- |
| **Annual sun exposure**  **to trunk and limbs** | **10 SED** | **30 SED** | **90 SED** |
| **NB-UVB MED on buttock J/cm^2^ (SED in parentheses)** | **Number of NB-UVB exposures**  **(95% CI in parentheses)** | | |
| 0.2 (1.1) | 479 (295 - 492) | 210 (117 - 193) | 78 (39 - 71) |
| 0.3 (1.7) | 598 (372 - 579) | 262 (148 - 228) | 98 (56 - 79) |
| 0.35 (2.0) | 693 (437 - 618) | 304 (178 - 256) | 113 (67 - 94) |
| 0.4 (2.3) | 816 (554 - 686) | 358 (230 - 279) | 133 (84 - 101) |
| 0.5 (2.9) | 1218 (909 - 940) | 534 (370 - 382) | 199 (133 - 138) |
| 0.6 (3.4) | 1986 (1294 - 1762) | 871 (542 - 731) | 324 (187 - 259) |
| 0.7 (4.0) | 3541 (1916 - 3552) | 1552 (868 - 1536) | 578 (286 - 515) |
| 0.8 (4.6) | 6901 (3103 - 9628) | 3025 (1259 - 3825) | 1127 (447 - 1319) |
| 0.9 (5.1) | 14699 (4541 - 26541) | 6443 (1928 - 10182) | 2400 (946 - 3790) |
| 1.0 (5.7) | 34221 (9336 - 55443) | 15000 (3062 - 29520) | 5587 (1298 - 10841) |
